# Tensile Properties and Deformation of AISI 316L Additively Manufactured with Various Energy Densities

**DOI:** 10.3390/ma14195809

**Published:** 2021-10-04

**Authors:** Matias Jaskari, Sumit Ghosh, Ilkka Miettunen, Pentti Karjalainen, Antti Järvenpää

**Affiliations:** 1 Kerttu Saalasti Institute, University of Oulu, Pajatie 5, 85500 Nivala, Finland; antti.jarvenpaa@oulu.fi; 2 Materials and Mechanical Engineering, Centre of Advanced Steels Research, University of Oulu, P.O. Box 4200, 90014 Oulu, Finland; sumit.ghosh@oulu.fi (S.G.); ilkka.miettunen@oulu.fi (I.M.); pentti.karjalainen@oulu.fi (P.K.)

**Keywords:** 316L stainless steel, additive manufacturing, laser-powder bed fusion, energy density, defect structure, tensile properties

## Abstract

Additive manufacturing (AM) is an emerging fabrication technology that offers unprecedented potential for manufacturing end-to-end complex shape customized products. However, building products with high performance by AM presents a technological challenge. Inadequate processing parameters, fabrication environment or changes in powder properties may lead to high defect density in the part and poor mechanical properties. Microstructure, defect structure, and mechanical properties of AISI 316L stainless steel pieces, additively manufactured by the laser powder bed fusion method using three different volume energy densities (VEDs), were investigated and compared with those of a commercial wrought AISI 316L sheet. Scanning and transmission electron microscopies were employed for characterization of grain and defect structures, and mechanical properties were determined by tensile testing. It was found that the number of defects such as pores and lack of fusion in AM specimens did not affect the strength, but they impaired the post-uniform elongation, more significantly when processed with the low VED. Twinning was found to be an active deformation mechanism in the medium and high VED specimens and in the commercially wrought material in the later stage of straining, but it was suppressed in the low VED specimens presumably because the presence of large voids limited the strain attained in the matrix.

## 1. Introduction

Recently, additive manufacturing (AM) has become a popular method for prototype and small batch manufacturing. Complex designs and lean structures can be optimized already in part design [1,2] so that the geometries are optimized and that material is used only where necessary. Small batches or unique parts can also be fabricated relatively quickly, as the process from the design to a finished part only contains a few steps. Laser powder bed fusion (L-PBF) is one of the most prominent AM techniques that has gained high interest in the aerospace, defense, and energy industries during the past ten years [3]. In the L-PBF, powder material is deposited layer by layer and melted with heating by a laser. Austenitic stainless steels such as 316L are a material group commonly used in AM processing, which are suitable, e.g., for biomedical applications and high-performance parts because of their good mechanical and corrosion properties.

The L-PBF process is, however, relatively complex, and several variables from powder properties to process parameters and part geometry affect the part quality and properties achieved. Specimens manufactured by the L-PBF usually have a layered morphology consisting of features with different size scales consisting of overlapping melt pools with fine cellular substructure in the build direction [4,5]. The morphology of the grain structure depends on various factors, but the volume energy density (VED) is a commonly used parameter in comparisons. The formed melt track bead sizes vary due to the process parameters employed, and the laser power is reported to influence the melt track width and depth [6]. Crystallographic grain structure is also dependent on VED. Increasing the total energy input causes repeated melting, solidification, and pronounced undercooling of several layers, which lead to columnar grain growth [6].

In addition to microstructure, density and defects in the material have an important influence on the mechanical properties of L-PBF products, and numerous studies have been concentrated on minimizing the defects through process and material optimization, e.g., [6,7]. A theoretical density of 99.5% is considered to be a highly dense material [5,6]. The achievable density level for L-PBF 316L is relatively high, and densities close to 99.99% have been achieved. However, the method is susceptible to process variations, and thereby, the achieved density can easily decrease. A low VED increases the probability of lack of fusion type defects [7], whereas a high VED can cause keyhole type and gas absorption defects [8], as well as balling phenomena [9,10]. Especially, the lack of fusion type defects is known to be detrimental to fatigue properties, as the sharp features can work as pre-cracks in dynamic loading [7].

Achieving a high density with a maximal build rate enables more efficient usage of AM. For example, Sun et al. [11] reported over 70% increase in building rate while achieving nearly 100% density of AM 316L. On the other end of the spectrum is the development of faster processes for less critical parts and applications, where a certain number of defects would be allowed. Still, understanding the formation of these defects and their effect on properties is necessary before AM can be adopted in safety-critical environments [12].

AM 316L as-the built condition usually possesses approximately 10% higher strength and ductility in the horizontal (build plane) direction than the vertical (build) direction [13]. This anisotropy is a common feature for AM materials, and it is intensified by the epitaxial growth of columnar grains [14]. For as-built 316L, the yield strength (YS) is comparably high for stainless steel, around 400 MPa in the horizontal or build direction (BD) for L-PBF structures, and the strength is reported to result from the sub-structure [15,16]. Tensile strength is usually near that of the conventional wrought 316L, being around 700 MPa. Elongations are typically near that of the wrought material, but they are affected by the defect structure. Heat treatment can be employed as a post-processing treatment for residual stress relief or recrystallization annealing, as well as for optimization of the mechanical properties [17].

Even though the mechanical properties and deformation behavior of AM 316L is quite well described in the literature, the data are often dispersed, and the effect of large defects and defect structure is neglected. Further, the data are often acquired from as-built samples and lack the conventional stress-relief annealing that is usually done for additively manufactured (AMed) parts. In the current study, the effect of the level of VED on microstructure and defect formation in a 316L stainless steel in the L-PBF process and the effect of the microstructure and porosity on mechanical properties and deformation mechanisms were investigated using electron microscopy and tensile testing. Structures were AMed with parameters near the standard ones, and a conventional stress relief annealing cycle was used. This way the effect of material density, microstructure, and the deformation behavior could be emphasized so that they corresponded well to those in the conventional AM in industry.

## 2. Materials and Methods

**Figure 1:**
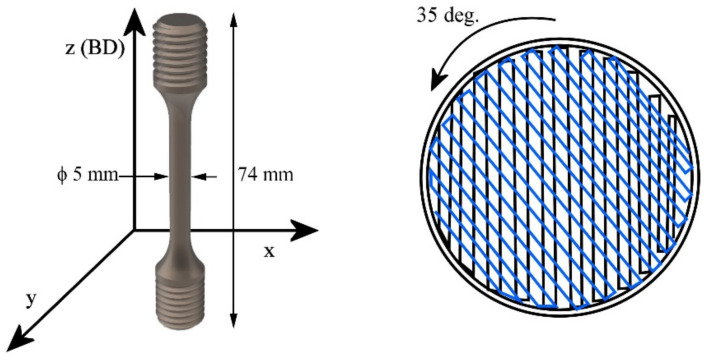
Schematic of building direction, tensile specimen, and build hatching plan. Laser scan track was rotated 35 degrees after each layer.

Material was AMed from a commercial AISI 316L powder, supplied by SLM (SLM Solutions, Lübeck, Germany). A commercial wrought AISI 316L bar with 25 mm diameter was used as a reference material. The chemical compositions were measured using an ARL 9800 XP Xray spectrometer (Thermo Fisher, Waltham, MA, USA), and they are listed in Table 1. In the table, the stacking fault energy (SFE) of the materials is also given, to be used later in discussion. For L-PBF processing of the specimens, three VED levels were chosen: 50.8, 79.2, and 84.3 J/mm^3^—called for simplicity as low, medium, and high-VED, respectively, by varying the laser power and scan speed. The VEDs were chosen to represent various types of densities and microstructures, being lower, near, or above the VED that is conventionally used in fabrication of AISI 316L with the used equipment. Specimens were fabricated using an SLM 280HL L-PBF (SLM Solutions, Lübeck, Germany) device with the parameters listed in Table 2. Specimens were built in the vertical direction so that the testing axis was perpendicular to the weld track, as illustrated in Figure 1. After fabrication, the specimens were stress-relief annealed in a muffle furnace (Nabertherm, Lilienthal, Germany) at 600 °C for 120 min, followed by slow cooling to room temperature in the furnace.

The VED was calculated using the equation

VED = P/vσt = [J/mm^3^], (1)

where P is the used laser power, v is the scan speed, σ is the hatch spacing, and t is the layer thickness.

Microstructures were examined from polished cross-sections cut in the XZ-plane (please refer to Figure 1 for the directions). The cut samples were first carefully ground and mechanically polished to mirror finish and electrochemically etched with nitric acid solution and 1.2 V DC. Etched specimens were then studied using a laser microscope (optical examination, Keyence VX-200, Keyence, Osaka, Japan) or a field-emission scanning electron microscope (SEM; Zeiss UltraPlus, Zeiss, Jena, Germany). For SEM examinations, a SE-detector with 5 kV acceleration voltage and 8 mm working distance was employed. For electron backscatter diffraction (EBSD, Oxford Instruments, Abingdon, UK), specimens ground to 600 Grit surface finish were electropolished to mirror finish using perchloric acid solution and 23 V DC. Employed acceleration voltage and working distance for EBSD were 15 kV and 11 mm, respectively. For TEM studies, focused ion beam milling (FIB; FEI Helios, Thermo Fisher, Waltham, MA, USA) was used to extract thin lamellas from both the base material and near fracture surface of a tensile specimen. Specimens were then examined with TEM (JEOL JEM-2200FS, JEOL, Tokyo, Japan) using 200 kV. Material density was measured by the Archimedes’ principle using ethanol as the measurement liquid, as well as from polished cross-sections of each studied sample.

Tensile tests were carried out at two strain rates of 0.008 and 0.0005 s^−1^ using as-built, threaded round tensile specimens built in the vertical direction, as illustrated in Figure 1. Specimen dimensions were designed according to standard EN ISO 6892-1, so that the used test diameter was 5 mm and total length 74 mm. The test and gauge lengths were 35 and 25 mm, respectively. M10 threaded ends were used as gripping heads. At least three specimens were tested in each case.

## 3. Results and Discussion

### 3.1. Additively Manufactured and Wrought Microstructures

**Figure 2:**
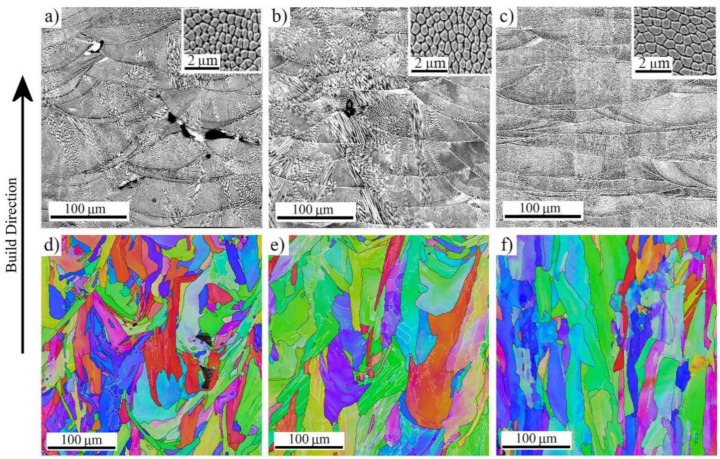
Laser optical and EBSD IPF images of 316L fabricated with (**a**,**d**) low-VED; (**b**,**e**) medium-VED; (**c**,**f**) high-VED. Inserts in a, b, and c display the average cell structure captured with SEM in the same XZ-direction.

**Figure 3:**
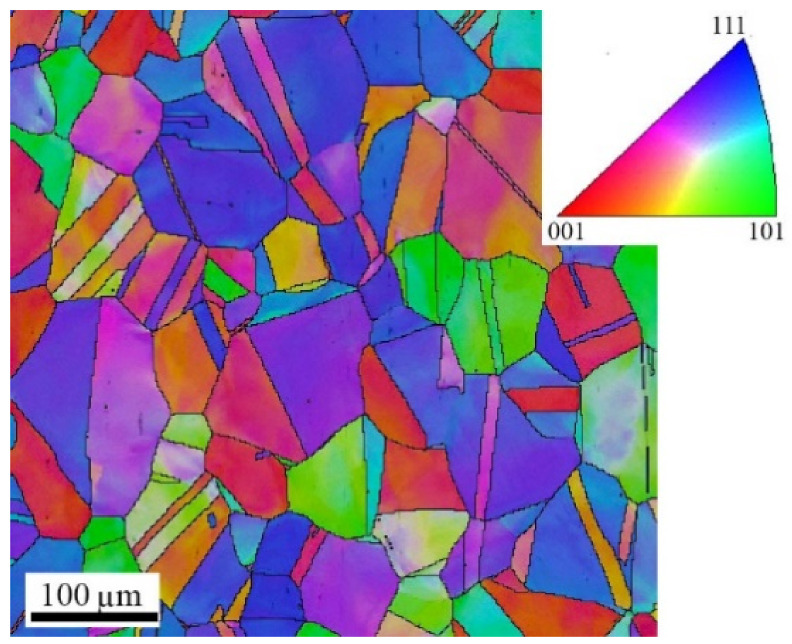
IPF image of the wrought 316L sheet microstructure consisting of coarse equiaxed austenite grains.

The microstructures of the specimens AMed at the three VEDs are shown in laser optical microscopy (LOM) and EBSD images in Figure 2. Overall bead size increased slightly with increasing the VED. The largest difference was observed in the melt pool width, which increased from 155 to 285 µm from the low-VED to high-VED specimen. Substructure measurements revealed the average cell sizes as 0.40, 0.52, and 0.85 µm for the low, medium, and high VED specimens, respectively. As noticed from the orientation distributions in EBSD images, the grain orientations were almost randomly distributed in the low-VED specimen, and the grain shape was very irregular. However, the grain shape altered towards a more columnar type in the medium- and high-VED structures. This is a result of the epitaxial growth due to the remelting and rapid cooling of the several top-most manufactured layers during the fabrication process. The occurrence of columnar grain growth could also be concluded from the aspect ratio TD/BD in Table 2, as it decreased with increases in the VED from 0.67 to 0.42. Additionally, the measured grain sizes increased with increases in the VED from 10.5 to 15.3 µm. The wrought 316L bar consisted of almost equiaxed coarse austenitic grains, the average grain size being 62 µm. Some orientation rotation was present due to rolling and annealing, as seen in Figure 3.

### 3.2. Density and Defect Structure

**Figure 4:**
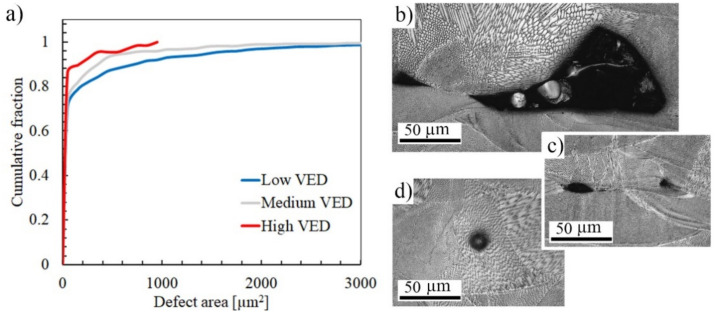
(**a**) Defect size distributions as cumulative fractions in L-PBF AISI 316L and (**b**) typical large lack-of-fusion type void. (**c**) Lack of fusion defect with sharp characteristics and (**d**) a round pore formed by gas bubble.

Density measurements (results in Table 3) and cross-sectional images revealed a clear reduction from 99.6 down to 91.9% in the material density with decreasing VED. Numerous defects existed especially in the low-VED structure. As seen from the size distribution in Figure 4a, the defect sizes varied from small to relatively large in the low-VED specimen, and the distribution shifted towards smaller cross-sectional sizes in the higher VED structures. The largest defect size observed was 109, 65, and 21 µm for low-, medium-, and high-VED specimen, respectively. The shape of the defects varied in all structures because in addition to relatively large pores (Figure 4b), narrow lack-of-fusion type voids were detected, as seen in Figure 4c. In the high-VED specimen, some gas pores were also present (Figure 4d). Typically, these structures can also contain very narrow boundary defects and pre-cracks between the melt pool boundaries.

### 3.3. Mechanical Properties

Tensile tests were conducted with two strain rates of 0.008 (higher) and 0.0005 s^−1^ (lower), and the results are listed in Table 3. At the higher strain rate, the YS and ultimate tensile strength (UTS) were found to be nearly independent of the VED value, around 480 and 785 MPa, respectively, within the parameter window used in the experiments. The same trend could also be seen in the experiments with the lower strain rate. Although the YS of the AMed structures was higher compared with that of the wrought 316L (390 MPa), the TS was lower (785 and 805 MPa) than that for the wrought structure (887 and 935 MPa) at the higher and lower strain rate, respectively. When comparing with strength values reported in the literature, the measured strength properties were of the same order, although the YS values could vary depending on the AM technology and post-treatment applied. For example, Pham et al. [19] reported a higher YS of 520 MPa for as-built L-PBF AM 316L, highlighting fine subgrains with high dislocation density and strong twinning-induced plasticity behavior. Barkia et al. [16] drew the same conclusion with their studies for a directly deposited 316L, where the substructure with high dislocation density and nano-sized precipitates was attributed to the strength properties. Suryawanshi et al. [20] also reported a high YS between 510 and 535 MPa of an as-built L-PBF 316L, YS depending slightly on the building strategy. Logically, the present heat treatment at 600 °C for 120 min has a slight decremental effect on strength properties, and the present YS agrees better with the YS of PBF 316L (around 440 MPa) annealed at 700 °C, measured by Ronneberg et al. [21]. Relatively low annealing temperature affects the material mostly by recovery, leaving the strength properties close to those of the as-built properties, although the annihilation of dislocations during heat treatment affects the YS.

The elongations of low- and medium-VED specimens were inferior compared with those of the high-VED structure and wrought material, the uniform elongation (UE) being about 0.27 and 0.38, respectively. Total elongations (TEs) followed the same trend, and they were related to the structure densities. Decreasing the strain rate to 0.0005 s^−1^ had a noticeable positive effect on the ductility, and UE and TE increased for all structures, UE up to 0.48 for the high-VED specimen. For instance, Röttger et al. [22] have reported that SLM-built 316L components have a higher strength than cast components, but they have a lower elongation at fracture. The lower elongation values correlate with the porosity and can be attributed to microstructural defects such as pores, cracks, or binding defects.

**Figure 5:**
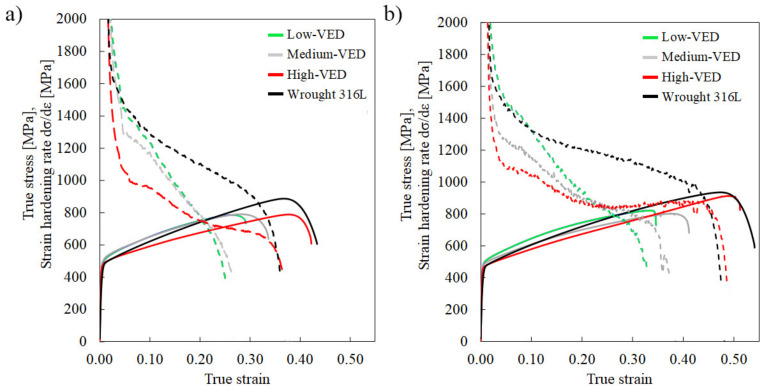
True stress vs. true strain (solid lines) and strain hardening rate curves (dashed lines) of the studied 316L strained at the (**a**) higher and (**b**) lower strain rate, respectively.

Strain hardening rate (SHR) curves shown in Figure 5 reveal the low SHR of AMed structures at both the strain rates, and they are consistent with the data of Wang et al. [23]. The low-VED and wrought structures possessed slightly higher SHR in early stages of the straining, but the SHR of the low-VED specimen decreased quickly to the low level of the medium- and high-VED specimens. Even though all SHR values were low, decreasing below 1000 MPa after 0.1 true strain, the level of the SHR of the high-VED specimen stayed constant (about 900 MPa) or even increased again after 0.25 strain (red curve in Figure 5b). This suggests that some additional strengthening took place in the later stages of tensile straining. A similar trend seems to exist also at the higher strain rate (Figure 5a), although the slope of the SHR curve remained declining at all strains.

### 3.4. Strained Microstructures

**Figure 6:**
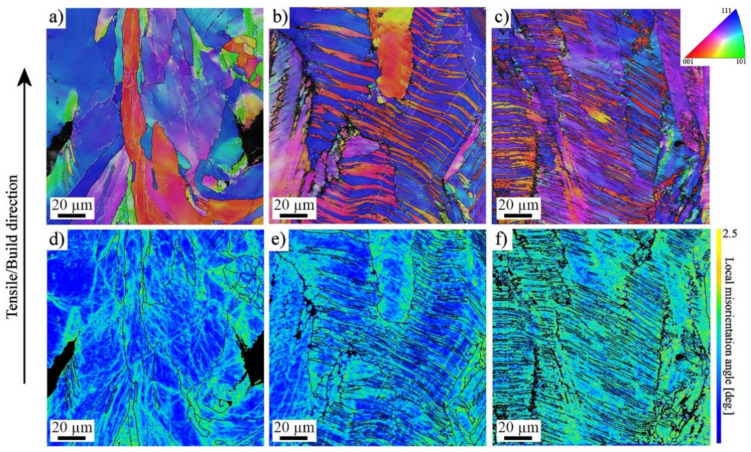
IPF and KAM maps of L-PBF 316L strained at the higher strain rate until UE, fabricated with the (**a**,**d**) low-; (**b**,**e**) medium-; (**c**,**f**) high-VED.

**Figure 7:**
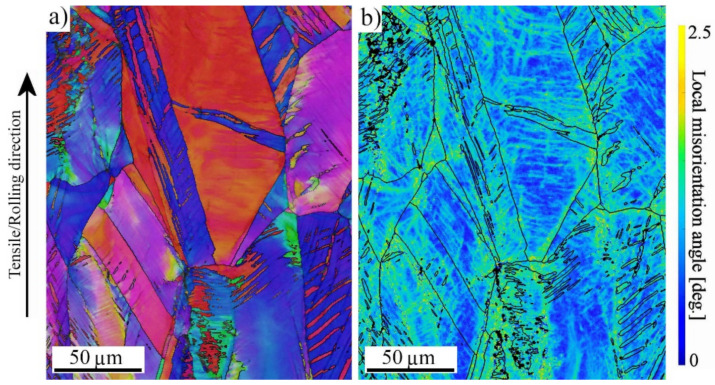
(**a**) IPF and (**b**) KAM maps of wrought 316L, strained at the higher strain rate. Twinning is present mainly in [111]-orientated grains.

To further clarify the active deformation mechanisms during tensile straining, specimens strained until fracture were examined with EBSD. Figure 6 shows the IPF and Kernel average misorientation (KAM) maps taken from a uniform elongation area of specimens strained at the higher strain rate. For the low-VED specimen formation of slip bands, an orientation gradient was present (Figure 6a), but in addition to them, also twinning became evident in the medium- and high-VED specimens (Figure 6b,c). Twins accommodated the same [001]-type orientation along the BD as the grains without twins. Twinning took place mostly in [111]-orientation grains, which is consistent with the findings of Wang et al. [23], showing that the twinning tendency was governed by the crystal orientation. Furthermore, they calculated the effective SFE to be the lowest in the [111]-orientation grains, which promoted the occurrence of twinning. This also agrees with experiments conducted, for instance by Pham et al. [19], where the deformation twinning was found to be dominant in columnar L-PBF 316L structures during plastic straining. Woo et al. [24] measured in situ the effective SFE during the plastic deformation of an AM 316L, and they noticed it ranging from 46 to 21 mJ/m^2^, depending on the applied strain. This means that even if the stress-free SFE of the AM 316L steel is higher than the ideal one for twinning, the effective SFE will decrease during straining, and twinning can be enhanced. The KAM image in Figure 6d indicates some strain localization in regions adjacent to grain boundaries and around the pores in the low-VED specimen, but in the medium- and high-VED structures, the local misorientation concentrated either in twins or [001]-oriented grains (Figure 6e,f). The behavior was similar in the wrought material in Figure 7, where twinning was also present in an [111]-orientated grain. However, as seen in Figure 6b,c, the existing twins are very wide and relatively sparsely located, so they did not enhance the SHR as effectively as the mechanical twins did in twinning-induced plasticity (TWIP) steels. Some additional examinations were performed at 0.10 strain, and no twins were detected in that stage. Thus, it can be concluded that twins were formed during the later stages of straining, so that their total effect on elongation properties was minor, only affecting the post-uniform straining behavior. Consistently, the SHR curve of the high-VED structure (Figure 5b) becomes horizontal at the true strain of 0.25, which suggests that twinning might be activated in that stage. However, no such behavior can be seen for the medium-VED structure in spite of twins being detected at the latest stage of straining. In the wrought structure, SHR is clearly higher than in the AMed structures, so the defect density may also affect the level of SHR. In the low-VED structure, only few twins could be seen near the pores (Figure 6a) but not in the matrix. Röttger at al. [22] reported that the elongation was correlated with the defect density, which in the present instance explains the lowest elongation of the low-VED structure, which contained the most numerous defects. Consequently, because the strain level remained lower in the low-VED specimen than in the medium- and high-VED specimens, twinning also remained less, or was practically absent.

**Figure 8:**
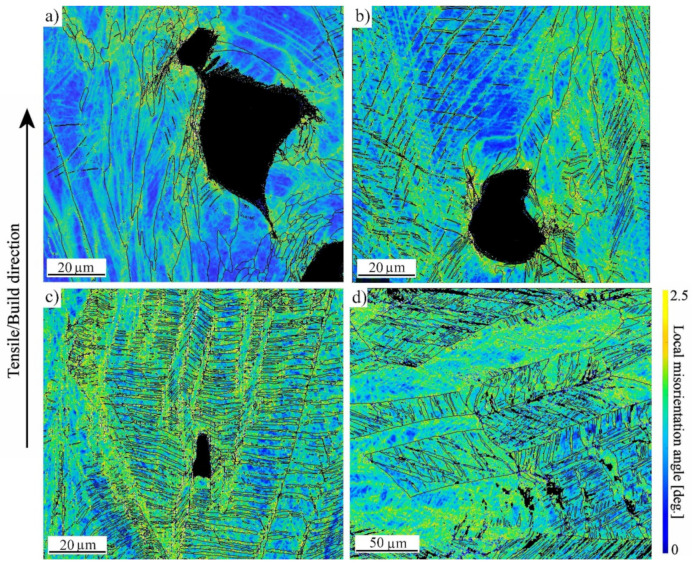
KAM maps of 316L strained at lower speed, fabricated with (**a**) low; (**b**) medium; (**c**) high VED; and (**d**) wrought 316L. In image (**d**) black areas correspond to α′-martensite.

Voids also affected the straining behavior at the low strain rate of 0.0005 s^−1^, as evident from the KAM images in Figure 8. For the medium- and high-VED specimens as well as for the wrought 316L strains seem to localize near pores or in twins. Instead, for the low-VED specimen, local strains were concentrated in regions adjacent to big pores, but the strains in the matrix remained relatively lower. This is also seen in the lowest UE and TE values (Table 4). The deformation mechanism seems to be the same in both the AMed and wrought specimens, although some deformation-induced α’-martensite formation could be detected in the wrought material, even though the calculated SFE (Table 1) was well above the commonly known martensite transformation limit of 20 J/mm^2^ [25]. Inhibiting the adiabatic heating during straining by using the low strain rate enables more efficient twinning and even the phase transformation, as temperature rise is known to increase the SFE [26,27] and thereby favor deformation by dislocation glide.

### 3.5. Fracture Behavior

**Figure 9:**
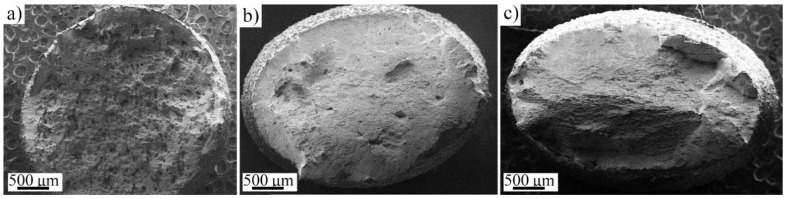
Macroscopic SE-images of (**a**) low-; (**b**) medium- and (**c**) high-VED samples strained at the lower strain rate.

**Figure 10:**
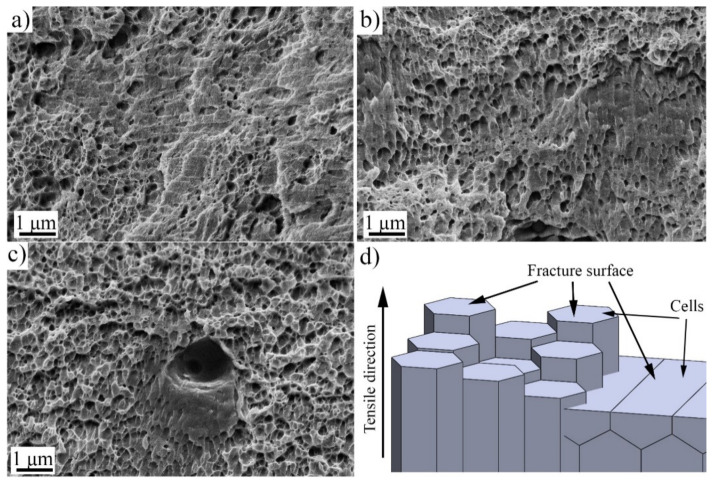
Higher magnification SE-images taken from the fracture surfaces of (**a**) low-; (**b**) medium; and (**c**) high-VED specimens strained at lower strain rate and (**d**) illustration of the fracture surface.

Fracture surfaces of the AMed specimens strained at the low strain rate were investigated using SEM. Macroscopic images of fractured surfaces are shown in Figure 9, indicating that the medium- and high-VED specimens exhibited anisotropic deformation behavior (Figure 9b,c), but in the low VED specimen (Figure 10a) the fracture surface was rougher, and the cross-sectional shape was almost spherical. The anisotropy of L-PBF AISI 316L, in comparison with BD and other directions, is a well-known phenomenon [13,28], and texture is proposed to be one of the main factors for this [29]. However, in the present instance, the anisotropy seems to also exist in the X- and Y-direction. In a macroscopic scale, the low-VED specimen had undergone fracture almost without necking, whereas the medium- and high-VED samples exhibited more ductile-type fracture and necking behavior. The effect of pore size and density for as-built AM 316L has been studied by Röttger et al. [22], who argued that even a relatively low porosity content can lead to premature fracture if the distance between the pores is short or the pore size is large. This condition seems to exist here for the low-VED structure. Crack propagation does not take place perfectly perpendicular to the stress direction, but the crack is frequently deflected by microstructural defects, which leads to a fracture surface with a high roughness. In the present experiments, a rough surface and minimal necking is seen for the low-VED specimen in Figure 9a resulting from a high density of defects.

When the specimens were examined with a higher magnification, shown in Figure 10, all studied fracture surfaces showed features of the ductile fracture with dimples. However, in the low-VED sample, areas of very shallow dimples or flat regions were common, as seen in Figure 10a. The size of the dimples seems to correspond roughly to the cellular substructure, i.e., they are of submicron size. Thus, it may happen that the material fractures through the cells or along cell boundaries, depending on their orientation to tensile direction, as schematically illustrated in Figure 10d. Still further studies are needed to verify this. The substructure is known to have chemical inhomogeneity along the cell boundaries [30], which could in turn affect the deformation behavior.

### 3.6. Associated Failure Micro-Mechanisms

**Figure 11:**
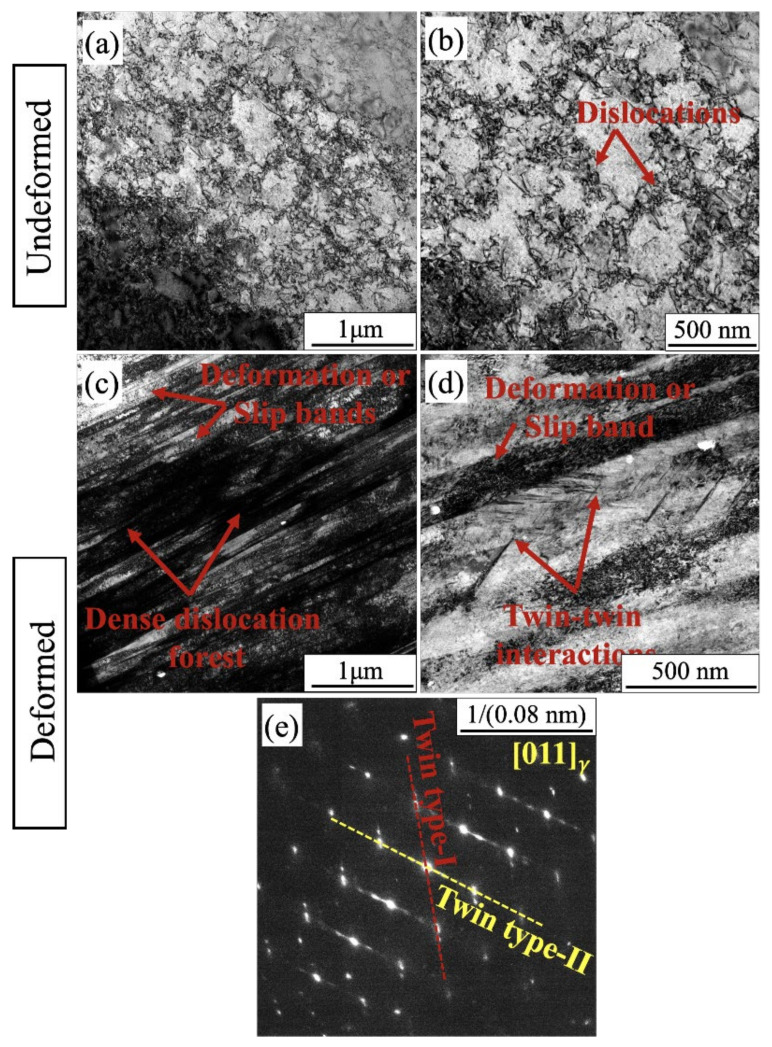
(**a**) Bright field (BF) TEM image of the microstructure adjacent to failure region; (**b**) magnified view of the selected area; (**c**) BF image showing dense dislocation forest and deformation bands; (**d**) magnified view of selected location; (**e**) selected area electron diffraction of the corresponding location from area in (**d**).

To study further the fracture and deformation behavior, the high-VED specimen strained with the higher strain rate was examined with a TEM and compared with the undeformed specimen. Figure 11a,b shows TEM bright field low and high magnification images of the undeformed as-built and stress relief-annealed specimen, respectively. The undeformed specimen exhibited low density of dislocations and typical dislocation-network structure of the AMed AISI 316L specimen, typical to the cellular substructure [31]. Figure 11c,d displays TEM bright-field micrographs adjacent to the tensile fracture surface of the high-VED specimen. Plastic deformation involving dislocation slip as well as twinning is observed close to the fracture location (Figure 11c,d), consistent with the EBSD results (Figure 6). The twins seen in the uniform elongation area of the high-VED specimen in Figure 6c were sparsely distributed. The relatively darker contrast within the deformation regions suggests the presence of high dislocation density due to distortion associated with the straining before failure. This agrees with the findings of Yin et al. [32]. They, however, observed twinning to be an active deformation mechanism already in the early stages of plastic strain enhancing the SHR. In the present case, the low- and medium-VED specimens did not show any enhancement of the SHR even at larger strains, but it was seen for high-VED specimens after a strain of 0.25 (Figure 5). The interactions between twins are also visible in Figure 11d. Tensile straining activates multiple twinning systems in the FCC matrix and twin–twin interactions occur. The corresponding selected area electron diffraction (SAED) pattern taken along [011]γ zone axis showed an intersection of two sets of twin planes (type-1 and type-2). The formation of deformation twins during the straining decreased the mean free path of dislocations and the blocking motion of dislocations and thereby enhanced the strain hardening, although in the present case this effect was not so prominent from a mechanical properties point of view, as twinning only happened in the later stages of plastic straining. However, UE and TE of the high-VED specimen were distinctly higher than those of the low-VED and medium-VED specimens, almost equal to those of the wrought steel (Table 4). The contributions of twinning and defect density are hard to distinguish, however.

**Figure 12:**
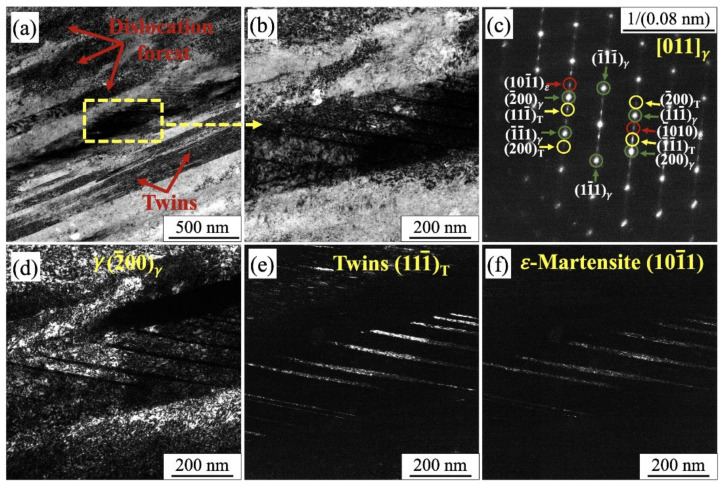
(**a**) Bright field TEM image of the microstructure adjacent to failure region; (**b**) magnified view of the selected location; (**c**) SAED pattern of the corresponding location; (**d**–**f**) dark field images of corresponding SAED spot.

Figure 12a depicts a close view of a location of twin–twin interaction and presence of dense dislocation forest within a twin lamella region. Figure 12b shows a magnified view of specific location as marked in Figure 12a, and the corresponding SAED is in Figure 12c. A simultaneous diffractions of deformation twin and ε-martensite are identified with the zone axis [011]γ//[11–20]ε, as indexed in Figure 12c. Dark field images corresponding to matrix (−200), twin (11–1), and ε-martensite (10–11) spots are displayed in Figure 12d–f, respectively. This indicates that the ε-martensite phase is formed as thin platelets on {111} twin planes. Transformation of austenite to ε-martensite during plastic straining of AISI 316L and 304 is also reported in the literature [33], and the orientation relationship corresponds well with the findings of Huang et al. [34].

## 4. Conclusions

In this study, microstructure, defect structure, and tensile properties and behavior of an L-PBF AISI 316L stainless steel, manufactured using three different (low, medium, high) volume energy densities (VEDs) were investigated and compared with those of a commercial wrought AISI 316L sheet. Two strain rates were applied in tensile testing. As conclusions, some highlights can be listed as follows:

- Microstructures and density of the additively manufactured AISI 316L varied according to the used VED. Increase in VED caused substructure coarsening and columnar growth but also an increase in achieved density. The type and distributions of defects observed in the studied structures varied from narrow lack-of-fusion defects to large voids in the low-VED structure, whereas the distribution shifted towards smaller cross-sectional sizes in the higher VED structures. The largest defect size observed was 109, 65, and 21 µm for low-, medium-, and high-VED specimen, respectively.
- The value of VED had no influence on the yield and tensile strength within the applied parameter window, but the elongation was increased with increases in the VED, with the high-VED specimen being almost equal to that of the wrought steel. Decreasing the strain rate enhanced the elongation of both the additively manufactured and wrought structures. All samples showed ductile fracture behavior, but the necking was almost inhibited in the low-VED structure, obviously affected by the high defect density.
- Twinning was found to be an additional deformation mechanism in the last stages of plastic straining in the medium- and high-VED structures as well as in the wrought material, regardless of the strain rate, and it was also found to be orientation-dependent. In the low-VED specimen, twinning was only found locally near the relatively large defects. Presumably, this was a result of low accumulated strain in the matrix before fracture.
- Some ε-martensite was found to form adjacent to the twins in the high-VED structure during necking. This indicates that the effective stacking fault energy of the steel is reduced during straining to become low enough for the formation of ε-martensite and twins, though only in the necking stage in the present steel.

## Figures and Tables

**Table 1:** Chemical composition and stacking fault energy of additively manufactured and commercial wrought AISI 316L.

Material	Element [wt.%]										SFE [mJ/m^2^] [18]
	C	Cr	Ni	Mn	Mo	Si	Ti	Nb	N	Fe	
L-PBF	0.02	17.7	12.9	0.6	2.5	0.7	0.01	0.005	0.09	Bal.	27
Wrought	0.02	17.0	10.3	1.5	2.0	0.1	0.04	0.02	0.04	Bal.	33

**Table 2:** Fabrication parameters for AISI 316L specimens.

ID	Volume Energy Density [J/mm^3^]	Laser Power [W]	Scan Speed [mm/s]	Hatch Spacing [µm]	Layer Thickness [µm]
low-VED	50.8	160	875	120	30
medium-VED	79.4	190	800	100	30
high-VED	84.3	220	725	120	30

**Table 3:** Grain size, aspect ratio, and the measured density for AMed and wrought AISI 316L.

VED [J/mm^3^]	Grain Size [µm]			Melt Pool Size [µm]		Aspect Ratio	Density
	TD	BD	Equivalent Diam.	Width	Height	TD/BD	%
50.8	9.3	13.8	10.5	155	54	0.67	91.9
79.4	15.9	23	15.3	202	67	0.69	94.6
84.3	14.3	33.7	15.2	285	62	0.42	99.6
Wrought	39.1	52.3	61.8	-	-	0.75	100

**Table 4:** True stress-true strain values of studied 316L structures. VED = volume energy density, YS = yield strength, UTS = ultimate tensile strength, UE = uniform elongation and TE = total elongation.

VED [J/mm^3^]	0.008/s				0.0005/s			
	YS [MPa]	UTS [MPa]	UE	TE	YS [MPa]	UTS [MPa]	UE	TE
50.8	481	786	0.26	0.29	479	821	0.33	0.35
79.4	489	790	0.28	0.34	478	805	0.37	0.41
84.3	475	788	0.38	0.42	460	834	0.48	0.51
Wrought	390	887	0.38	0.43	382	935	0.47	0.54

## Data Availability

Data is contained within the article.
